# Brain White Matter Tract Integrity and Cognitive Abilities in Community-Dwelling Older People: The Lothian Birth Cohort, 1936

**DOI:** 10.1037/a0033354

**Published:** 2013-08-12

**Authors:** Tom Booth, Mark E. Bastin, Lars Penke, Susana Muñoz Maniega, Catherine Murray, Natalie A. Royle, Alan J. Gow, Janie Corley, Ross D. Henderson, Maria del C. Valdés Hernández, John M. Starr, Joanna M. Wardlaw, Ian J. Deary

**Affiliations:** 1 Centre for Cognitive Ageing and Cognitive Epidemiology and Department of Psychology, University of Edinburgh, Edinburgh, Scotland; 2 Centre for Cognitive Ageing and Cognitive Epidemiology and Brain Research Imaging Centre, Division of Neuroimaging Sciences, University of Edinburgh, and Scottish Imaging Network, A Platform for Scientific Excellence (SINAPSE) Collaboration; 3 Centre for Cognitive Ageing and Cognitive Epidemiology and Department of Psychology, University of Edinburgh, and Scottish Imaging Network, A Platform for Scientific Excellence (SINAPSE) Collaboration; 4 Department of Psychology, University of Edinburgh; 5 Centre for Cognitive Ageing and Cognitive Epidemiology, University of Edinburgh, and Psychology, School of Life Sciences, Heriot-Watt University, Edinburgh, Scotland; 6 Centre for Cognitive Ageing and Cognitive Epidemiology and Geriatric Medicine Unit, University of Edinburgh; 7 Centre for Cognitive Ageing and Cognitive Epidemiology and Department of Psychology, University of Edinburgh

**Keywords:** cognitive ability, tractography, white matter integrity, bifactor model

## Abstract

***Objective:*** The present study investigates associations between brain white matter tract integrity and cognitive abilities in community-dwelling older people (*N* = 655). We explored two potential confounds of white matter tract−cognition associations in later life: (a) whether the associations between tracts and specific cognitive abilities are accounted for by general cognitive ability (*g*); and (b) how the presence of atrophy and white matter lesions affect these associations. ***Method:*** Tract integrity was determined using quantitative diffusion magnetic resonance imaging tractography (tract-averaged fractional anisotropy [FA]). Using confirmatory factor analysis, we compared first-order and bifactor models to investigate whether specific tract-ability associations were accounted for by *g*. ***Results:*** Significant associations were found between *g* and FA in bilateral anterior thalamic radiations (*r* range: .16−.18, *p* < .01), uncinate (*r* range: .19−.26, *p* < .001), arcuate fasciculi (*r* range: .11−.12, *p* < .05), and the splenium of corpus callosum (*r* = .14, *p* < .01). After controlling for *g* within the bifactor model, some significant specific cognitive domain associations remained. Results also suggest that the primary effects of controlling for whole brain integrity were on *g* associations, not specific abilities. ***Conclusion:*** Results suggest that *g* accounts for most of, but not all, the tract−cognition associations in the current data. When controlling for age-related overall brain structural changes, only minor attenuations of the tract−cognition associations were found, and these were primarily with *g*. In totality, the results highlight the importance of controlling for *g* when investigating associations between specific cognitive abilities and neuropsychology variables.

Cognitive ability is associated with many important life outcomes. The retention of cognitive functioning is of particular importance to successful aging (Deary et al., 2009), with a growing body of research exploring the associations between cognitive ability and measures of white matter integrity in the brain (see Madden et al., 2012, for a recent review). Recent research has suggested that communicative white matter pathways are an important aspect of neurostructural foundation of cognitive ability (Deary, Penke, & Johnson, 2010).

Diffusion tensor imaging (DTI) is a widely used technique for studying brain connectivity (Sullivan & Pfefferbaum, 2006), providing biomarkers of white matter integrity, in particular fractional anisotropy (FA), which measures the directional coherence of water molecule diffusion. In normal aging, FA shows a gradual mean decrease, indicative of decreasing white matter tract integrity (Wozniak & Lim, 2006). Herein, we focus on studies that have used DTI tractography as this method was applied in the current study (see the Method section).

DTI tractography studies of associations between white matter tract integrity and cognitive ability in older people have provided inconsistent findings (Madden et al., 2012). Higher FA in the genu of corpus callosum has been associated with working memory (Davis et al., 2009; Zahr, Rohlfing, Pfefferbaum, & Sullivan, 2009; Voineskos et al., 2012), while significant associations have been found between integrity of the right uncinate fasciculus and spatial working memory (Davis et al., 2009), and the left cingulum and performance on verbal paired associates (a test of verbal declarative memory) and executive function (Davis et al., 2009; Sasson, Doniger, Pasternak, Tarrasch, & Assaf, 2012). Penke et al. (2012), using a subsample of the participants from the current study (*n* = 420), found that general factors of white matter integrity derived from three brain imaging biomarkers (i.e., FA, longitudinal relaxation time [T_1_], and magnetization transfer ratio) significantly predicted general intelligence, and that this prediction was fully mediated by processing speed. Though Penke et al. provided strong support for the associations between white matter tract integrity and cognitive ability, specific tract associations were not considered.

Inconsistency in associations between white matter tracts and cognitive abilities makes it difficult to provide substantive theoretical explanations for the associations. For example, the parieto-frontal integration theory (P-FIT) has been proposed as an integrative framework for understanding the associations between the brain and intelligence (Jung & Haier, 2007). P-FIT suggests that the arcuate fasciculus, which forms part of the superior longitudinal fasciculus, may be particularly important in understanding tract−cognitive ability associations (Jung & Haier, 2007; Colom et al., 2009; Turken et al., 2008). However, as can be seen from the above brief review of studies in aging samples, the arcuate fasciculus has not been consistently associated with cognitive ability.

In the current study, we explored two possible methodological reasons for the inconsistent findings in aging samples. First, the studies documented above assessed both general and specific (e.g., verbal, spatial, memory) cognitive abilities. From such studies, it is not clear the extent to which correlations between white matter tract integrity and specific cognitive abilities are accounted for by general cognitive ability. This is of interest because each cognitive test score will comprise a proportion of variance that is attributable to specific ability, a proportion that is attributable to general ability, as well as a proportion of error variance (see Deary et al., 2010, for discussion in the context of neuroscience). The importance of partitioning this variance in neuroimaging studies of cognitive ability has been previously noted by a number of authors (e.g., Colom & Thompson, 2011; see also Chen, West, & Sousa, 2006, for a more general discussion), but no studies to date have been able to do so using highly robust methods.

Figures 1 and 2 graphically depict how test score variance is decomposed based on the methods applied to study the associations of cognitive ability and neuroimaging measures. Figure 1a depicts perhaps the most common situation, in which a single cognitive test score is associated with a neuroimaging measure (e.g., Davis et al., 2009; Sasson et al., 2012; Zahr et al., 2009). The test score comprises general ability variance, specific ability variance, and error variance. In such circumstances, it is not possible to know which aspect of score variance is driving the correlation with the external measure. It is also important to note that, if researchers choose to sum a number of standardized scores (*z* scores) from individual tests into a single composite, the same effect (as is depicted in Figure 1a) occurs, and variance cannot be separated.[Fig-anchor fig1][Fig-anchor fig2]

To try and estimate the extent to which specific ability variance associates with neuroimaging measures, some authors (e.g., Colom et al., 2009; Haier et al., 2009; Tang et al., 2010) have regressed a general cognitive ability score (e.g., a sum score or factor score) on individual test scores, and have associated the resultant residual with neuroimaging measures (see Figure 1b). Although such methods partial out general ability variance, the residual term still consists of both specific ability and error variance, thus the association remains muddied.

Both situations described above are based on analyses of single variables, be they individual test scores or summed composites. In recent years, it has become increasingly common to apply exploratory (EFA) and confirmatory factor analysis (CFA), and the more general structural equation modeling (SEM) framework to investigate associations between cognitive ability and neuroimaging measures (see Kievit et al., 2012; Penke & Deary, 2010, for discussions). In SEM, a measurement model is specified in which multiple cognitive tests are used to estimate latent cognitive ability factors based on the common variance across test scores. Simultaneously, the estimated latent factors can be associated with the neuroimaging measures of interest. A primary advantage of SEM approaches is that latent variables are error-free (Bollen, 1989), because they are estimated from only common variance between tests. Error variance is explicitly modeled in SEM as a residual term on observed variables (test scores).

SEM is, therefore, highly useful in accounting for one source of variance—error variance—which may confound associations between cognitive and neuroimaging measures. However, some specifications of measurement models fail to separate general cognitive ability variance from specific ability variance. For example, in a first-order factor model (see Figure 2a), common variance associated with both the specific ability and the general cognitive ability is conflated in the latent variable. Once again, it is not clear whether the association with neuroimaging variables is driven by general or specific abilities. Figure 2a depicts only a single latent variable; however, the above statement remains true when multiple first-order latent variables are modeled.

A possible solution to this problem is the application of bifactor models. Bifactor modeling has been advocated as the best method for simultaneously measuring both specific and general cognitive abilities (Gignac, 2008; Schmiedek & Li, 2004; Brunner, Nagy, & Wilhelm, 2012; see Reise, Moore, & Haviland, 2010; Yung, Thissen, & McLeod, 1999, for technical details on the estimation of bifactor models). Figure 2b depicts the decomposition of test score variance within a bifactor model. Here, a latent general cognitive ability factor is estimated based on all test scores, while specific ability latent factors are estimated from a subset of test scores hypothesized to measure a specific ability. In Figure 2b, for example, Tests 1−3 may be arithmetic tests, whereas Tests 4 and 5 may be verbal tests. Thus, bifactor modeling provides a framework within which test score variance can be decomposed into its constituent parts, which can then be associated with external variables. To our knowledge, no studies of white matter tract integrity have applied a bifactor modeling approach.

In the current study, we compared the results of first-order and bifactor models within the SEM framework, to help understand the extent to which brain white matter tract associations with specific cognitive abilities are caused by cognitive variation unique to that ability or general cognitive ability. The reliable identification of specific and general cognitive ability factors, and their associations with neuroimaging biomarkers, may be of particular importance in aging samples (Schmiedek & Li, 2004), given that specific abilities, such as processing speed, memory, reasoning, and spatial skills, start to decline much earlier than experience-based specific abilities, such as vocabulary and knowledge (Salthouse, 2011).

A second potential methodological issue the current study sought to explore is to what extent are any cognitive associations with specific white matter tracts owed to more general aspects of age-related brain degeneration. In general, the aging brain displays both gray and white matter atrophy, and white matter lesions (Anderton, 2002) as well as accumulating microstructural changes that are not sufficient to show as overt lesions on conventional imaging. These features of the aging brain have been suggested to cause disconnections in cognitive networks (Bullmore & Sporns, 2009), and to be predictive of cognitive ability in later life (Deary, Leaper, Murray, Staff, & Whalley, 2003). Herein, we investigated whether these general aspects of brain integrity impact on tract−cognitive ability associations, and whether these attenuations are stronger for general or specific cognitive abilities.

## Method

### Participants

Participants were drawn from the Lothian Birth Cohort, 1936 (LBC1936), a longitudinal study of cognitive aging. Most participants took part in the Scottish Mental Survey, 1947, at about age 11 years, and were residents of Edinburgh and its surrounding area (the Lothians) at recruitment to Wave 1 of the study at about age 70 years. Protocols for recruitment, testing, and brain magnetic resonance imaging (MRI) are reported in detail elsewhere (Deary et al., 2007; Deary, Gow, Pattie, & Starr, 2011; Wardlaw et al., 2011).

From the original 1,091 participants in Wave 1 (*M*_age_ = 69.5 years, *SD* = 0.8), 866 participants returned in Wave 2 (*M*_age_ = 72.5 years, *SD* = 0.7), of which 700 provided some usable data from structural and diffusion MRI. In the present study, a cutoff of 25% was applied for missing data, which resulted in 39 subjects being removed for missing MRI data and one for missing cognitive ability data. Further, subjects were removed from analysis if they scored below 24 on the Mini-Mental State Examination (Folstein, Folstein, & McHugh, 1975), because a score below 24 is often considered an indicator of possible pathological cognitive impairment. Five subjects were removed based on this criterion. A total sample of 655 was used in the current study.

### Ethics Approval

Ethics permission for the LBC1936 study protocol was obtained from the Multi-Centre Research Ethics Committee for Scotland and the Lothian Research Ethics Committee. All research was carried out in compliance with the Declaration of Helsinki.

### Cognitive Ability Measures

The current analyses used 18 cognitive ability subtest scores. Full details of the cognitive tests have been published previously (Deary et al., 2007; see also supplemental materials, Table A).

Briefly, we used seven subtest scores (Logical memory immediate recall and Logical memory delayed recall, Verbal paired associates immediate recall and Verbal paired associates delayed recall, Digit span backward, and Spatial span forward and Spatial span backward) from the Wechsler Memory Scale III (Wechsler, 1998b); five subtests (Block design, Matrix reasoning, Digit symbol coding, Symbol search, Letter−number sequencing) from the Wechsler Adult Intelligence Scale III (Wechsler, 1998a); the National Adult Reading Test (Nelson & Wilson, 1991), the Wechsler Test of Adult Reading (Holdnack, 2001), verbal fluency (Lezak, 2004), an inspection time task of visual information processing (Deary et al., 2004), and simple and four-choice reaction time tasks (Deary, Der, & Ford, 2001).

### Image Acquisition

Full details of the image acquisition can be found in Wardlaw et al. (2011). In brief, participants underwent whole brain structural and high angular resolution 2-mm isotropic voxel diffusion MRI (seven T2- and 64 diffusion-weighted [b = 1,000 s/mm^2^] axial single-shot spin-echo echo-planar imaging volumes) on a Signa Horizon HDxt 1.5T clinical scanner (General Electric, Milwaukee, Wisconsin) using a shelf-shielding gradient set (maximum gradient = 33 mT/m), and an eight-channel phased-array head coil. The structural MRI included T2-, T2*-, and fluid attenuated inversion recovery (FLAIR)-weighted scans, and a high-resolution T1-weighted volume scan.

### Tract Segmentation

The diffusion MRI data were preprocessed using FSL tools (FMRIB, Oxford, England) to extract the brain, remove bulk patient motion and eddy current−induced artifacts, and generate parametric maps of FA. Underlying connectivity data were generated using bedpostx and probtrackx with the default settings of a two-fiber model per voxel, and 5,000 probabilistic streamlines with a fixed separation of 0.5 mm between successive points (Behrens, Johansen-Berg, Jbabdi, Rushworth, & Woolrich, 2007).

Twelve tracts of interest were identified using probabilistic neighborhood tractography, a novel approach for automatic and reproducible tract segmentation (Clayden, Storkey, & Bastin, 2007), as implemented in the TractoR package for fiber tracking analysis (Clayden et al., 2011). Briefly, this method works by segmenting the same fasciculus-of-interest across a group of subjects from single seed-point tractography output by modeling how individual tracts compare to a predefined reference tract in terms of their length and shape (Clayden et al., 2007). In practice, multiple native space seed points are placed in a cubic neighborhood of voxels (typically 7 × 7 × 7) surrounding a seed point transferred from the center-of-the-reference tract, which is defined in standard space, with the tract that best matches the reference chosen from this group of “candidate tracts.” Tracts assessed were the genu and splenium of corpus callosum, and bilateral anterior thalamic radiations, rostral cingulum bundles, arcuate, uncinate, and inferior longitudinal fasciculi. Tract masks generated by probabilistic neighborhood tractography were overlaid on the FA parametric maps and tract-averaged values of these biomarkers, weighted by the connection probability, determined for each tract in every subject.

To ensure that the segmented tracts were anatomically plausible representations of the fasciculi of interest, a researcher (S. M. M.) visually inspected all masks blind to the other study variables and excluded tracts with aberrant or truncated pathways. In general, probabilistic neighborhood tractography was able to segment the 12 tracts of interest reliably (see Clayden, Storkey, Muñoz Maniega, & Bastin, 2009) in the majority of subjects, with tracts that did not meet quality criteria, such as truncation or failing to follow the expected path, ranging from 0.3% for the splenium of corpus callosum to 16% for the left anterior thalamic radiation, with a mean of 5%. (Failures in tract segmentation are typically caused by underlying tractography errors in bedpostx and probtrackx resulting from finite image resolution, small registration mismatches in the component diffusion MRI volumes, and measurement noise.) From the point of view of substantive investigations, the 12 tracts represent a good balance between projection, commissural, and association fibers, which connect a wide variety of brain regions.

### Structural MRI Volumetric Analysis

Brain tissue volumes were measured blind to participant information using a validated multispectral segmentation tool, MCMxxxVI (Wardlaw et al., 2011; Valdés Hernández, Ferguson, Chappell, & Wardlaw, 2010; see also http://sourceforge.net/projects/bric1936), from the coregistered structural MRI data. The tissue compartments measured were intracranial volume (ICV; i.e., all soft tissue structures inside the cranial cavity, including the brain, dura, cerebrospinal fluid [CSF], and venous sinuses); total brain tissue volume (i.e., brain tissue volume without the superficial or ventricular CSF); CSF (i.e., all CSF inside the cranial cavity, including the ventricles and superficial subarachnoid space); and white matter lesion volumes. Because MCMxxxVI does not distinguish hyper- and hypointense areas of cerebromalacea, due to old cortical or subcortical infarcts or lacunes, from white matter lesions and CSF, respectively, these areas were masked out from the respective binary masks by thresholding the FLAIR sequence using a region-growing algorithm from Analyze, Version 10.0 (see http://www.analyzedirect.com/Analyze). Where stroke lesions were confluent with white matter lesions, the boundary between the two was determined by comparison with the contralateral hemisphere and neuroradiological knowledge.

### Visual White Matter Lesion Rating

White matter lesion burden was also rated from T2- and FLAIR-weighted sequences by an expert neuroradiologist using the Fazekas scale. Lesions were coded based on whether they were located in subcortical or periventricular white matter, and the individual scores were summed to give an overall lesion rating (Wardlaw et al., 2011).

### Statistical Analysis

We performed two primary analyses. First, associations between tract integrity and specific cognitive abilities were compared, with and without controlling for *g*, using first-order and confirmatory bifactor models. In the second, we considered whether atrophy and white matter lesion load, the latter assessed using both volume measurements and visual rating scores, accounted for the associations between tract integrity and cognitive ability. The input data for all models were standardized residuals after regressing age, sex, and handedness on tract integrity measures, as well as age in days, and sex on each cognitive test.

### First-Order Versus Bifactor Models

In the first set of analyses, EFA was initially applied to identify the appropriate number of first-order cognitive ability factors (specific abilities) from our battery of 18 tests. Next, CFA was used to estimate both first-order and bifactor structural models for the cognitive tests, based on the results of the exploratory analysis. We defined a first-order model as containing only specific cognitive abilities as factors. The bifactor model contains both a general cognitive ability factor and specific cognitive ability factors. Structural equation models were estimated in which FA from each of the 12 segmented tracts was correlated with the cognitive ability factors in both models.

#### Exploratory factor analysis

EFA was conducted using maximum likelihood estimation and oblique Equamax rotation. The number of factors to extract was determined using parallel analysis (Horn, 1965) and minimum average partial (Velicer, 1976), using the “psych” package in R.2.13.2 (Revelle, 2013).

#### Confirmatory factor analysis

The exploratory factor solution was tested in both first-order (see Figure 3) and bifactor (see Figure 4) models. In the first-order model, specific cognitive abilities are modeled by factor loadings on subtest scores. The specific cognitive ability factors are allowed to correlate, but no general cognitive factor is included. In the bifactor model, each subtest score is loaded on both its specific factor and a general cognitive ability factor, thereby accounting for the variance in performance on that test that is general, not due to specific cognitive factors.[Fig-anchor fig3][Fig-anchor fig4]

In both the first-order and the bifactor models, a number of correlated residuals were included. In a confirmatory factor model, residuals contain the proportion of variance not accounted for by the latent construct, namely, unique and error variance. The battery of cognitive tests used in the current study includes subtests for which two scores have been retained, for example, Verbal paired associates immediate recall and delayed recall. Such scores will share test-specific variance, which would not be expected to be explained by the latent construct. See the Results section for full details on the residual correlations included.

#### Structural equation models

In the structural models, the 12 tract-averaged FA values were included and allowed to correlate with each cognitive ability factor. The tract-averaged FA values were allowed to correlate, following the empirical findings of a general integrity factor using a subsample of the LBC1936 data (Penke et al., 2012).

### Covarying for Total Brain Atrophy and White Matter Lesion Load

In the second set of analyses, the first-order and bifactor models were reestimated including total white matter lesion rating score (Fazekas), white matter lesion volume as a percentage of ICV, and brain atrophy—calculated as: atrophy = (1 – [total brain tissue volume/ICV]) × 100—as covariates in the model. The aim of the second analysis was to ask whether specific white matter tract–cognitive ability associations were attenuated by controlling for more general measures of brain integrity.

### Structural Equation Model Estimation and Evaluation

All models were estimated using full information maximum likelihood (FIML) estimation in Mplus, Version 6.0 (Muthén & Muthén, 2010). FIML was used because a small proportion of missing data was present (see the Results section for details). FIML is considered to be one of the most robust missing data techniques (Enders & Bandalos, 2001).

Model fit was evaluated based on recommendations from the Monte Carlo simulation studies of Hu and Bentler (1998, 1999), and a review by Schermelleh-Engel, Moosbrugger, and Muller (2003). We adopted cutoff points of 0.05 or less for the standardized root mean square residual (SRMR), 0.06 or less for the root mean square error of approximation (RMSEA), and .95 or greater for the Tucker−Lewis index (TLI) and comparative fit index (CFI). If a model displays appropriate levels of fit, it is considered to be a good representation of the data, and the researcher can consider substantive interpretations of parameter estimates.

## Results

### Descriptive Statistics

Descriptive statistics for the cognitive test results, tract-averaged FA values, and covariates are presented in Table 1. The greatest proportion of variance missing from any individual variable is 16.0% (*n* = 105), for the left anterior thalamic radiation. Across all variables, the proportion of missing data was low. Simple reaction time score and Inspection time total correct responses displayed the greatest levels of skew or kurtosis (4.09 and 3.82, respectively). However, on inspection of the histograms, these deviations from normality were considered small. All other variables displayed close to normal distributions.[Table-anchor tbl1]

### First-Order Versus Bifactor

Results of minimum average partial analysis suggested that two factors should be retained from the analysis of the 18 cognitive tests, whereas parallel analysis suggested seven factors should be retained. All factor solutions with between two and seven factors were tested. The seven-, six-, and five-factor solutions all contained underidentified factors (fewer than three indicators), and/or Heywood cases (implausible loadings > 1.00). Therefore, these solutions were rejected. The four-factor solution (see supplemental materials, Table B1) was retained because it represented the most psychologically interpretable solution retaining the greatest number of specific cognitive factors. The four-factor solution also remained stable across different forms (i.e., geomin, FC-parsimax, and oblimin) of oblique rotations. Factor consistency across rotational methods is generally considered a marker of a robust factor solution (Sass & Schmitt, 2010). The four factors were labeled knowledge, verbal declarative memory, processing speed, and nonverbal reasoning.

Next, we tested the EFA solution as a first-order CFA model (see Figure 3) and a bifactor CFA model (see Figure 4). Across both models, three correlated residuals were included between Logical memory immediate recall and delayed recall, Simple reaction time mean and Choice reaction time mean scores, and Digit span backward and Letter−number sequencing. Though the inclusion of a greater number of correlated residuals would have improved model fit, it also would have resulted in identification problems in the bifactor model.

Both first-order (see Figure 3) and bifactor (see Figure 4) confirmatory models showed acceptable-to-excellent levels of fit across all indices. The specific cognitive ability factors in the first-order model correlated significantly and positively (range: .43−.76; *M* = .56).

In the bifactor model, retaining all correlated residuals from the previous model (see Figure 4), the factor loadings of each subtest on *g* were generally moderate to large (> .40), with the exception of Simple reaction time mean score (−.27), Spatial span forward (.32) and Spatial span backward (.38), and Inspection time total correct responses (.38). The average general factor loading was .51.

Table 2 presents the results when all 12 tract-averaged FA measurements were included in each of the models. In the discussion that follows, we focus on the raw associations. Given the current sample size, a significance level of *p* < .05 and 80% power, the current study is powered to identify associations of approximately ± .11 and greater. Further, applying a Bonferroni correction to the first-order and the bifactor models resulted in corrected *p* values of .0011 and .0008, respectively. As a result, we considered all associations significant to *p* < .001 to be robust to multiple comparisons, and values at *p* < .01 to be highly indicative, given the conservative nature of Bonferroni corrections.[Table-anchor tbl2]

In the first-order factor model (see Figure 3), which contains only specific cognitive abilities, a large number of significant tract associations are found between posterior-frontal tracts, especially with processing speed and nonverbal reasoning (see Table 2). A number of smaller significant associations (<.11) are also seen, again in posterior-frontal tracts, with knowledge and verbal declarative memory.

In the bifactor model (see Figure 4), in which *g* is controlled for, most of the significant associations with specific cognitive ability factors are markedly attenuated, and become nonsignificant (see Table 2). These results suggest that, in the current battery of tests, the associations between specific factors of cognitive ability and white matter tract integrity are largely driven by *g*, and not by separable specific cognitive ability variance.

As can be seen in Table 2, the strongest associations with *g* are found for bilateral uncinate fasciculi (left = 0.19, right = 0.26; *p* < .001) and anterior thalamic radiations (left = 0.16, *p* < .01; right = 0.19, *p* < .001). A small number of associations with specific cognitive abilities remain significant after controlling for *g*. In the bifactor model, the right uncinate fasciculus is significantly associated with knowledge (−0.16, *p* < .01). The left inferior longitudinal fasciculus (0.16, *p* < .001) and right anterior thalamic radiation (0.14, *p* < .05) are both associated with processing speed. The right uncinate fasciculus (−0.14, *p* < .05) is associated with nonverbal reasoning. The left arcuate fasciculus (−0.10, *p* < .05) is associated with verbal declarative memory.

### Covarying for Atrophy and White Matter Lesion Load

Next, we reestimated both the first-order and the bifactor models using input data residualized for whole brain integrity variables (see Table 2, columns 4 and 7 labeled Residuals). The results for the bifactor model suggest that most attenuation of associations were small, with all parameter changes at the second decimal place, and the greatest change in estimate being .08. For a number of associations, particularly with *g*, this resulted in estimates becoming nonsignificant. However, the strongest associations between specific tracts and *g* across models remained significant, namely, the right anterior thalamic radiation (0.12, *p* < .05), and the left (0.13, *p* < .05) and right (0.22, *p* < .001) uncinate fasciculus.

Further, it is also of interest that the residual attenuations on specific ability associations are generally greater in the first-order model than in the bifactor model. This suggest that much of the attenuation in tract−cognitive associations is attributable to *g*, because when this variance is separated in the bifactor model, the greatest attenuations are seen in *g*, not specific factor associations.

## Discussion

The results of the current study lead to three main conclusions. First, we provide further evidence that failure to control for *g* when investigating the associations between specific cognitive abilities and neuroimaging biomarkers could result in misleading, spurious, or inflated associations with the specific cognitive factors. Second, integrity in a large number of white matter tracts, primarily the uncinate fasciculus and anterior thalamic radiation, were associated with general cognitive ability, *g*, in our aging sample. However, a small number of associations with specific tracts remained, suggesting further robust analyses of specific associations would be beneficial. Third, the results suggest that, despite loss of brain structural integrity, that is, increased atrophy and white matter lesion load, associations between white matter tract integrity and cognitive ability are independent of these general indicators of brain structural decline.

In the current sample, higher general cognitive ability was significantly associated with greater white matter tract integrity in both right and left uncinate fasciculi and anterior thalamic radiations. In the limited research published to date on individual tract−cognitive ability associations in aging samples, neither of these two tracts has commonly been associated with *g*. However, Zahr et al. (2009) found significant age effects for the uncinate fasciculus when comparing small samples of young (*n* = 12; *M*_age_ = 25.5 years) and older (*n* = 12; *M*_age_ = 77.7 years) participants.

Across all individual tracts, the right uncinate fasciculus showed the greatest number of significant associations with both specific and general cognitive ability, supporting previous findings in younger samples (Yu et al., 2008). The right uncinate fasciculus is larger than the left, leading some to suggest greater connectivity and information flow between the right fronto-temporal regions it connects (Highley, Walker, Esiri, Crow, & Harrison, 2002). The number of significant associations found in the current study may therefore be a reflection of the greater connectivity of the right uncinate fasciculus.

Outside of the associations with *g*, the strongest bilateral association between any tract and cognitive ability was seen for the inferior longitudinal fasciculus and processing speed. This in part confirms prior findings of Davis et al. (2009), yet there remains much uncertainty as to the functional role of the inferior longitudinal fasciculus (Ashtari, 2012). Clearly, the current finding of a specific association requires replication, but the large sample and association after controlling for general cognitive ability suggest that further studies on speed of processing may be fruitful.

The importance of estimating specific cognitive abilities, controlling for *g*, was demonstrated in comparing the results from the first-order and the bifactor models. In the first-order model, a large number of the significant associations were found for processing speed and nonverbal reasoning, but were attenuated and became nonsignificant when *g* was controlled for in the estimation of specific ability factors using a bifactor model. Clearly, the associations of the specific abilities with white matter tract integrity were driven, at least in part, by the variance in test scores associated with general cognitive ability, not specific abilities.

The current study demonstrates the utility of bifactor modeling in aging samples (Brunner et al., 2012; Schmiedek & Li, 2004) to control for general cognitive ability in the associations between narrow level-specific abilities and neuroimaging variables (Colom & Thompson, 2011; Gignac, 2008; Chen, West, & Sousa, 2006). This is an important extension to past studies, which have generally taken one of two approaches to controlling for *g*, either by regressing out a sum score for *g* from sum scores for specific abilities (e.g., Colom et al., 2009), or by using a hierarchical factor analytic procedure, such as the Schmid−Lieman transformation, to extract factor scores for both *g* and specific abilities (e.g., Gläscher et al., 2010). The bifactor approach has a number of distinct advantages, most notably the ability to simultaneously estimate associations between criterion variables and specific and general cognitive ability factors, and the robust nature of the estimates based on latent constructs free from measurement error. Further, the bifactor model has a number of methodological advantages, such as being free from the proportionality constraints present in higher-order models and methods such as the Schmid−Lieman transformation (Schmiedek & Li, 2004).

**Figure 1 fig1:**
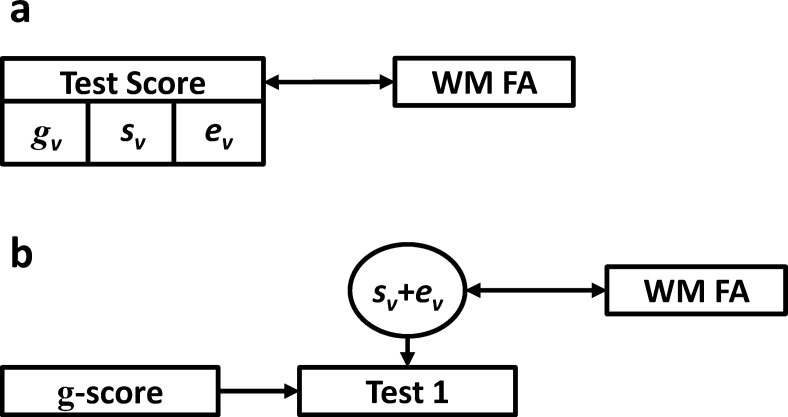
Diagrammatic representations of variance decomposition in two methods for estimating the association between cognitive ability and neuroimaging variables. A simple correlation between an individual cognitive test and sum score (a). Controlling for a total *g* or IQ score on a single test or sum score (b). Rectangles = observed variables; circles = latent or residual variables; single-headed arrows = direct paths; double-headed arrows = correlations; WM FA = white matter fractional anisotropy; *g*_*v*_ = general cognitive ability variance; *s*_*v*_ = specific cognitive ability variance; *e*_*v*_ = error variance.

**Figure 3 fig3:**
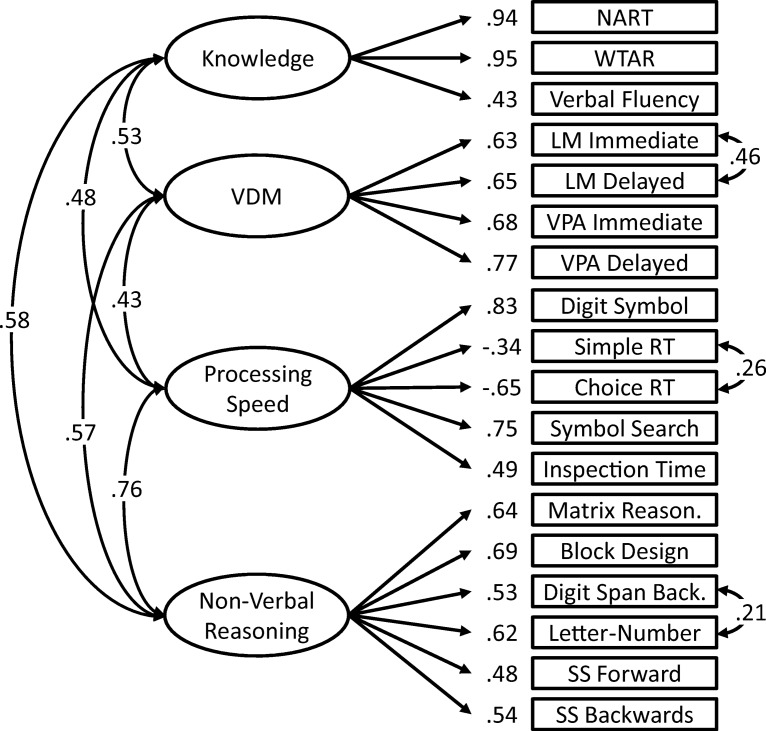
Measurement model for the first-order model. VDM = verbal declarative memory; NART = National Adult Reading Test; WTAR = Wechsler Test of Adult Reading; LM = Logical memory; VPA = Verbal paired associates; RT = reaction time; SS = Spatial span. Model Fit: χ^2^(126) = 417.99, *p* < .001; comparative fit index = 0.95; Tucker−Lewis index = 0.93; root mean square error of approximation = .059, 95% confidence interval [.053, .066]; standardized root mean square residual = 0.058.

**Figure 4 fig4:**
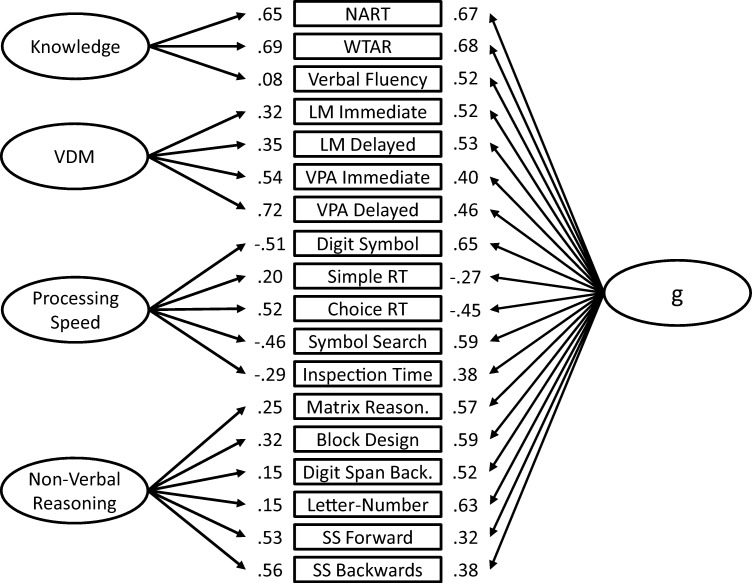
Measurement model for the bifactor model. VDM = verbal declarative memory; NART = National Adult Reading Test; WTAR = Wechsler Test of Adult Reading; LM = Logical memory; VPA = Verbal paired associates; RT = reaction time; SS = Spatial span. Model Fit: χ^2^(114) = 315.68, *p* < .001; comparative fit index = 0.96; Tucker−Lewis index = 0.95; root mean square error of approximation = .052, 95% confidence interval [.045, .059]; standardized root mean square residual = 0.044.

Our findings conform to previous suggestions from the literature (e.g., Colom & Thompson, 2011), and may go some way to explaining the variability of tract−cognitive ability associations found across studies. Commonly in neuroimaging studies of cognitive ability, researchers use single subtests or small batteries of subtests either to measure *g* or to measure specific abilities. These batteries are often sum-scored, and not measured as latent constructs using SEM (see Figure 1). When single tests are measured, it is not possible to determine the extent to which associations are driven by *g*, or specific abilities, because any individual test will vary in the level to which it measures *g* and specific abilities (Major, Johnson, & Bouchard, 2011). Thus, in any individual study, it may not be immediately apparent exactly which cognitive ability factor is being measured, and how much effect *g*, when not explicitly measured, is exerting on the associations found. If researchers are interested in associations between specific abilities and external constructs, we recommend gathering data on multiple subtests.

Consideration of the results of the models controlling for whole brain integrity (i.e., global atrophy and white matter lesion load) further emphasizes the importance of controlling for *g*. In the first-order model, attenuation in associations between tracts and specific cognitive ability factors was present for all specific ability factors, suggesting whole brain integrity is significantly associated with each of these factors. However, when controlling for *g* in the bifactor model, especially in the case of verbal declarative memory, processing speed, and fluid ability, the attenuations became much smaller, and the larger effects were seen in the *g* associations with specific tracts, suggesting that whole brain integrity more strongly influences *g*, not specific abilities.

### Study Limitations

There are a number of limitations to the current study. First, the specific cognitive ability factors within the bifactor model were identified by a limited number of individual test scores. In an ideal case, more individual tests would have been included in the battery to ensure over identification of these factors. Second, a number of studies (e.g., Haier, Jung, Yeo, Head, & Alkire, 2005; Tang et al., 2010) have suggested sex differences in tract−cognitive ability associations. In the current study, we chose not to investigate sex differences, but to control for variance in tests due to sex. Future research may consider whether the patterns of association found here are consistent across the sexes. Third, the observed lack of attenuation of the tract cognition associations by whole brain integrity variables may result from the measure of global atrophy being relatively insensitive to aging-related white matter damage, which typically affects subcortical structures and leads to ventricular enlargement. Finally, the present cohort was relatively healthy, and the results should not be taken to represent advanced stages of aging-related brain atrophy or white matter lesions.

### Conclusion

The current study has a number of major strengths. First, the availability of a large sample from a narrow range age cohort eliminates many of the potential confounds of age in cross-sectional studies. Second, by applying probabilistic neighborhood tractography, we were able to segment a large number of major white matter tracts and to measure tract-averaged FA in these pathways both reliably and automatically. Third, we extend the previous work of Penke et al. (2012) by focusing on specific tract associations using a larger sample. Fourth, we used both visual and computational measures of white matter lesion load because they provide different, but complementary, information on disease burden. Finally, we applied a broad battery of psychometric tests, which, in part due to our large sample size, allowed us to model latent cognitive ability factors within a structural equation model framework. The current study is, to our knowledge, the only study to combine bifactor modeling of general and specific cognitive abilities with tractography estimates of tract integrity. Collectively, we provide highly robust estimates of tract−cognitive ability associations.

The current study reports associations between white matter tract integrity and cognitive abilities in a large, age-homogeneous sample of relatively healthy older people. We found that the associations of specific cognitive abilities with external variables may be biased if researchers fail to account for *g*; that is, significant associations may not be due to a specific, but rather a general cognitive ability. However, once variance associated with *g* is controlled for, a number of specific ability−specific tract associations remained. Of import, this finding suggests the potential fruitfulness of further research based on robust methodologies in the investigation of specific cognitive abilities. Lastly, the results demonstrate that, in the current sample, controlling for atrophy and white matter lesion load does not alter tract−cognitive ability associations.

## Supplementary Material

10.1037/a0033354.supp

## Figures and Tables

**Table 1 tbl1:** Descriptive Statistics of Cognitive Ability Tests, Tractography Fractional Anisotropy Measures, and Covariates

Variable	No. missing	*M*	*SD*	Skew	Kurtosis
Cognitive ability					
WMS-III Logical memory immediate recall	1	45.88	10.18	−0.48	0.30
WMS-III Logical memory delayed recall	1	28.89	8.08	−0.56	0.26
WMS-III Verbal paired associates immediate recall	12	2.80	2.30	0.62	−0.66
WMS-III Verbal paired associates delayed recall	15	6.39	2.09	−1.26	0.57
WMS-III Spatial span forward	1	7.66	1.65	−0.09	−0.39
WMS-III Spatial span backward	2	7.08	1.60	−0.06	−0.10
Verbal fluency total score	1	43.47	12.68	0.23	0.14
National Adult Reading Test	1	34.57	7.86	−0.54	−0.10
Wechsler Test of Adult Reading	1	41.26	6.70	−0.93	0.61
Simple reaction time mean score	0	0.27	0.05	1.66	4.09
Choice reaction time mean score	0	0.65	0.08	0.89	1.77
Inspection time total correct responses	11	111.45	11.64	−1.16	3.82
WAIS-III Digit symbol	1	56.43	12.22	0.11	−0.20
WAIS-III Digit span backward	0	7.90	2.30	0.28	−0.20
WAIS-III Block design	2	34.26	9.98	0.47	0.13
WAIS-III Letter−number sequencing	0	11.01	2.99	0.28	0.41
WAIS-III Matrix reasoning	1	13.46	4.86	−0.10	−0.93
WAIS-III Symbol search	1	24.74	6.09	−0.32	0.78
Fractional anisotropy tractography					
Genu of corpus callosum	17	0.41	0.05	−0.07	−0.12
Splenium of corpus callosum	4	0.49	0.07	−0.30	0.61
Left arcuate fasciculus	27	0.45	0.04	−0.42	0.50
Right arcuate fasciculus	87	0.43	0.04	−0.29	0.72
Left anterior thalamic radiation	105	0.32	0.03	−0.10	0.25
Right anterior thalamic radiation	20	0.33	0.03	−0.31	0.54
Left rostral cingulum	23	0.44	0.05	−0.53	0.76
Right rostral cingulum	13	0.39	0.04	−0.61	1.84
Left uncinate fasciculus	93	0.33	0.03	−0.13	0.39
Right uncinate fasciculus	33	0.33	0.03	−0.25	0.47
Left inferior longitudinal fasciculus	4	0.40	0.05	−0.28	0.08
Right inferior longitudinal fasciculus	3	0.38	0.05	−0.40	0.18
Covariates					
Age	0	72.6	0.70	−0.00	−0.87
Atrophy (percent decline)	13	22.39	3.84	0.16	0.10
White mater lesion volume in ICV (%)	13	0.83	0.91	2.52	10.24
Fazekas total lesion rating score	6	2.45	1.14	0.83	0.83
		Male	Female		
Sex	0	345	310		
		Right	Left	Ambidextrous	
Handedness	0	614	38	3	
*Note.* WMS-III = Wechsler Memory Scale III UK; WAIS-III = Wechsler Adult Intelligence Scale III UK; ICV = intracranial volume.					

**Table 2 tbl2:** White Matter Tract Integrity and Cognitive Ability Correlations in the First-Order and Bifactor Models

Factors	First-order	95% CI	Residual (Δ)	Bifactor	95% CI	Residual (Δ)
Knowledge						
Genu corpus callosum	0.01	[−0.07, 0.09]	0.01 (0.00)	−0.03	[−0.13, 0.08]	0.00 (0.03)
Splenium corpus callosum	0.08	[0.00, 0.16]	0.06 (0.02)	−0.03	[−0.14, 0.07]	−0.03 (0.00)
Left arcuate fasciculus	0.02	[−0.06, 0.10]	0.00 (0.02)	−0.08	[−0.18, 0.02]	−0.07 (0.01)
Right arcuate fasciculus	0.02	[−0.06, 0.11]	0.01 (0.01)	−0.06	[−0.17, 0.04]	−0.06 (0.00)
Left anterior thalamic radiation	0.09*	[0.01, 0.18]	0.09* (0.00)	−0.03	[−0.14, 0.08]	0.00 (0.03)
Right anterior thalamic radiation	0.10*	[0.02, 0.18]	0.08* (0.02)	−0.04	[−0.14, 0.07]	0.00 (0.04)
Left rostral cingulum	0.05	[−0.03, 0.13]	0.05 (0.00)	−0.01	[−0.12, 0.09]	0.01 (0.00)
Right rostral cingulum	0.08*	[0.00, 0.16]	0.07 (0.01)	0.03	[−0.07, 0.14]	0.06 (0.03)
Left uncinate fasciculus	0.09*	[0.01, 0.17]	0.08 (0.01)	−0.05	[−0.15, 0.06]	−0.02 (0.03)
Right uncinate fasciculus	0.08	[0.00, 0.16]	0.08 (0.00)	−0.16**	[−0.26, −0.06]	−0.14** (0.02)
Left inferior longitudinal fasciculus	0.07	[−0.01, 0.15]	0.05 (0.02)	0.03	[−0.07, 0.13]	0.05 (0.02)
Right inferior longitudinal fasciculus	0.04	[−0.04, 0.11]	0.02 (0.02)	−0.01	[−0.12, 0.09]	0.00 (0.01)
Verbal declarative memory						
Genu corpus callosum	0.03	[−0.06, 0.12]	−0.01 (0.04)	0.04	[−0.05, 0.13]	0.04 (0.00)
Splenium corpus callosum	0.10*	[0.01, 0.18]	0.07 (0.03)	−0.01	[−0.10, 0.08]	−0.01 (0.00)
Left arcuate fasciculus	0.00	[−0.09, 0.09]	−0.07 (0.07)	−0.10*	[−0.19, −0.01]	−0.10* (0.00)
Right arcuate fasciculus	0.02	[−0.07, 0.12]	−0.03 (0.05)	−0.09	[−0.18, 0.00]	−0.09 (0.00)
Left anterior thalamic radiation	0.05	[−0.05, 0.14]	−0.01 (0.06)	−0.06	[−0.16, 0.04]	−0.06 (0.00)
Right anterior thalamic radiation	0.11*	[0.02, 0.20]	0.07 (0.04)	−0.02	[−0.10, 0.10]	0.02 (0.04)
Left rostral cingulum	0.01	[−0.08, 0.10]	−0.03 (0.04)	−0.07	[−0.16, 0.02]	−0.07 (0.00)
Right rostral cingulum	0.07	[−0.02, 0.16]	0.04 (0.03)	−0.07	[−0.16, 0.02]	−0.07 (0.00)
Left uncinate fasciculus	0.11*	[0.01, 0.20]	0.06 (0.05)	0.00	[−0.09, 0.10]	0.01 (0.01)
Right uncinate fasciculus	0.11*	[0.02, 0.20]	0.06 (0.05)	−0.05	[−0.14, 0.05]	−0.05 (0.00)
Left inferior longitudinal fasciculus	0.02	[−0.07, 0.10]	−0.03 (0.05)	−0.03	[−0.12, 0.06]	−0.01 (0.02)
Right inferior longitudinal fasciculus	0.02	[−0.07, 0.11]	−0.01 (0.03)	−0.07	[−0.16, 0.02]	−0.06 (0.01)
Processing speed						
Genu corpus callosum	0.04	[−0.05, 0.13]	−0.01 (0.05)	0.04	[−0.08, 0.17]	0.02 (0.02)
Splenium corpus callosum	0.13**	[0.05, 0.21]	0.07 (0.06)	0.05	[−0.08, 0.17]	0.01 (0.04)
Left arcuate fasciculus	0.15***	**[0.07, 0.24]**	0.05 (0.10)	0.10	[−0.02, 0.22]	0.03 (0.07)
Right arcuate fasciculus	0.13**	[0.04, 0.22]	0.04 (0.09)	0.07	[−0.06, 0.20]	0.01 (0.06)
Left anterior thalamic radiation	0.16***	**[0.07, 0.25]**	0.06 (0.10)	0.07	[−0.06, 0.20]	0.01 (0.06)
Right anterior thalamic radiation	0.23***	**[0.15, 0.31]**	0.15*** (0.08)	0.14*	[0.01, 0.26]	0.10 (0.04)
Left rostral cingulum	0.13**	[0.05, 0.22]	0.08 (0.05)	0.09	[−0.03, 0.22]	0.07 (0.02)
Right rostral cingulum	0.08	[−0.01, 0.16]	0.02 (0.06)	0.00	[−0.12, 0.13]	−0.03 (0.03)
Left uncinate fasciculus	0.13**	[0.04, 0.22]	0.03 (0.10)	−0.02	[−0.14, 0.11]	−0.08 (0.06)
Right uncinate fasciculus	0.13**	[0.04, 0.21]	0.06 (0.07)	−0.10	[−0.23, 0.02]	−0.13* (0.03)
Left inferior longitudinal fasciculus	0.19***	**[0.10, 0.27]**	0.08 (0.11)	0.16**	[0.04, 0.28]	0.08 (0.08)
Right inferior longitudinal fasciculus	0.15**	[0.06, 0.23]	0.08 (0.07)	0.11	[−0.02, 0.23]	0.06 (0.05)
Nonverbal reasoning						
Genu corpus callosum	−0.01	[−0.09, 0.08]	−0.05 (0.04)	−0.08	[−0.21, 0.05]	−0.08 (0.00)
Splenium corpus callosum	0.09	[0.00, 0.17]	0.04 (0.05)	−0.12	[−0.25, 0.01]	−0.13 (0.01)
Left arcuate fasciculus	0.07	[−0.02, 0.16]	0.00 (0.07)	−.10	[−0.23, 0.02]	−0.10 (0.00)
Right arcuate fasciculus	0.04	[−0.05, 0.14]	−0.02 (0.06)	−0.11	[−0.25, 0.02]	−0.12 (0.01)
Left anterior thalamic radiation	0.12*	[0.03, 0.21]	0.05 (0.07)	−0.09	[−0.23, 0.05]	−0.08 (0.01)
Right anterior thalamic radiation	0.19***	**[0.10, 0.27]**	0.14** (0.05)	−0.03	[−0.17, 0.12]	0.02 (0.05)
Left rostral cingulum	0.09*	[0.00, 0.18]	0.05 (0.04)	−0.03	[−0.16, 0.10]	−0.03 (0.00)
Right rostral cingulum	0.10*	[0.01, 0.19]	0.06 (0.04)	−0.00	[−0.13, 0.13]	0.01 (0.01)
Left uncinate fasciculus	0.16***	**[0.07, 0.25]**	0.10* (0.06)	−0.05	[−0.18, 0.09]	−0.04 (0.01)
Right uncinate fasciculus	0.18***	**[0.09, 0.26]**	0.13** (0.05)	−0.14*	[−0.27, −0.01]	−0.13 (0.01)
Left inferior longitudinal fasciculus	0.15**	[0.06, 0.23]	0.09 (0.06)	0.05	[−0.07, 0.18]	0.07 (0.02)
Right inferior longitudinal fasciculus	0.11**	[0.02, 0.20]	0.07 (0.04)	0.01	[−0.13, 0.16]	0.04 (0.03)
*g*						
Genu corpus callosum	^a^	–	–	0.03	[−0.07, 0.13]	−0.01 (0.04)
Splenium corpus callosum	^a^	–	–	0.14**	[0.04, 0.24]	0.11 (0.03)
Left arcuate fasciculus	^a^	–	–	0.12*	[0.02, 0.22]	0.05 (0.07)
Right arcuate fasciculus	^a^	–	–	0.11*	[0.00, 0.21]	0.06 (0.05)
Left anterior thalamic radiation	^a^	–	–	0.16**	[0.06, 0.27]	0.09 (0.07)
Right anterior thalamic radiation	^a^	–	–	0.19***	**[0.09, 0.29]**	0.12* (0.07)
Left rostral cingulum	^a^	–	–	0.10	[0.00, 0.20]	0.06 (0.04)
Right rostral cingulum	^a^	–	–	0.10	[0.00, 0.20]	0.06 (0.04)
Left uncinate fasciculus	^a^	–	–	0.19***	**[0.09, 0.29]**	0.13* (0.06)
Right uncinate fasciculus	^a^	–	–	0.26***	**[0.16, 0.35]**	0.22*** (0.04)
Left inferior longitudinal fasciculus	^a^	–	–	0.10*	[0.00, 0.20]	0.03 (0.07)
Right inferior longitudinal fasciculus	^a^	–	–	0.09	[−0.01, 0.20]	0.04 (0.05)
*Note.* All estimates are standardized. CI = confidence interval; Residual = correlations based on standardized residuals controlling for sex, age, handedness (tracts only), white matter lesion as a percentage of intracranial volume, Fazekas ratings of white matter lesions, and atrophy; (Δ) = the difference between raw and residualized associations.						
^a^ The first-order model contains no *g* associations because *g* was not included in this model.						
* *p* < .05. ** *p* < .01. *** *p* < .001. Values in boldface are significant after Bonferroni correction.						

**Figure 2 fig2:**
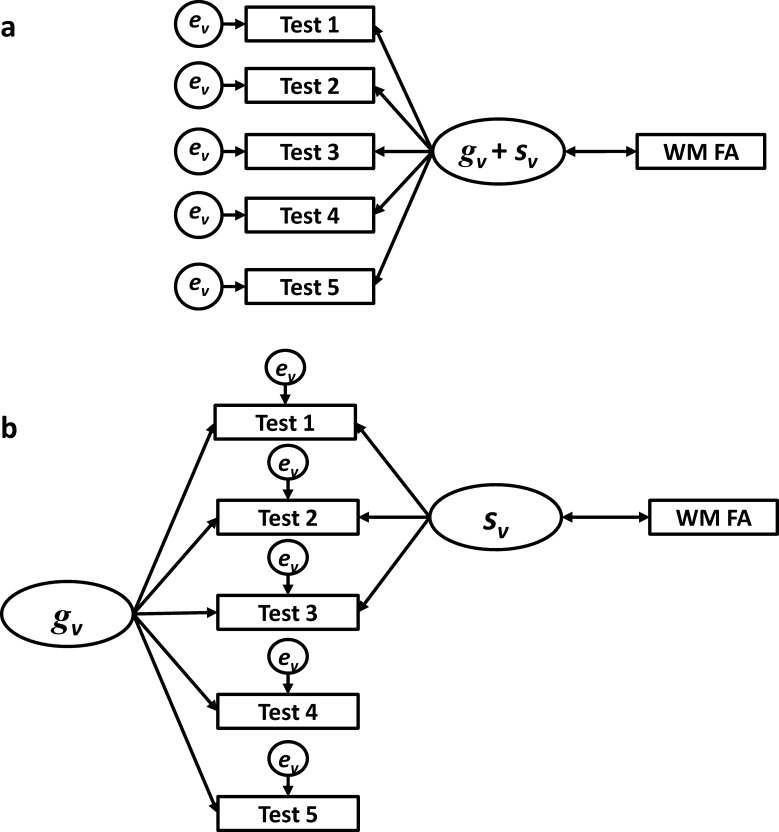
Diagrammatic representations of variance decomposition in two structural equation models for estimating the association between cognitive ability and neuroimaging variables. A first-order factor model (a). A bifactor model, including separate general and specific ability latent factors (b). Rectangles = observed variables; circles = latent or residual variables; single-headed arrows = direct paths; double-headed arrows = correlations; WM FA = white matter fractional anisotropy; *g*_*v*_ = general cognitive ability variance; *s*_*v*_ = specific cognitive ability variance; *e*_*v*_ = error variance.
