# Supplementary material for: Brain White Matter Tract Integrity and Cognitive Abilities in Community-Dwelling Older People: The Lothian Birth Cohort, 1936
Source: Neuropsychology. 2013 Aug 12;27(5):595–607. doi: 10.1037/a0033354 (PMC3780714; doi:10.1037/a0033354)
Supplement: Supplementary file 1 [file NEU-NEU2-Booth20121387-R-S1.doc]

**Online Supplementary Material**

**Brain White Matter Tract Integrity and Cognitive Abilities in Community-dwelling Older People: The Lothian Birth Cohort 1936**

**Corresponding Author:** Tom Booth ([tom.booth@ed.ac.uk](mailto:tom.booth@ed.ac.uk)). Centre for Cognitive Ageing and Cognitive Epidemiology, Department of Psychology, The University of Edinburgh, 7 George Square, Edinburgh, EH8 9JZ, UK.

**Supplementary Material A**

Further details of the cognitive ability tests used in the current analyses are provided below.

1. *Logical Memory (WMS-III^UK^)* tests verbal declarative memory and provides immediate and delayed recall scores. Participants are asked to recall two 25 item stories which are read aloud by the examiner. Immediate (3 recalls total possible score 75) and delayed (2 recalls total possible score 50) scores were used.
2. *Verbal Paired Associates (WMS-III^UK^)* tests learning and memory. Participants are read lists of unrelated word pairs and are asked to recall the second of the pair when given the first. Immediate and delayed scores were used.
3. *Digit Span Backward (WMS-III^UK^)* tests working memory, with participants asked to recall increasingly long strings of numbers backwards.
4. *Spatial Span (WMS-III^UK^)* tests non-verbal/spatial learning and memory. The participant observes a series of blocks being touched and then has to touch the blocks in the correct order. The procedure is repeated with participants required to touch the blocks in reverse order. Forward and backwards recall scores were used.
5. *Block Design (WAIS-III^UK^)* participants are asked to use blocks to reproduce a diagram of a specific design.
6. *Matrix Reasoning (WAIS-III^UK^)* tests abstract reasoning. Participants view an incomplete pattern within a matrix, and are required to select from a number of options which piece completes the matrix.
7. *Digit Symbol Coding (WAIS-III^UK^)* tests speed of information processing. The participant is required to enter a symbol according to a particular number-symbol code. Participants are given 2 minutes to complete as many items as possible.
8. *Symbol Search (WAIS-III^UK^)* tests speed of information processing. Participants are given two target symbols and have to decide (yes or no) whether either symbol appears in a row of symbols. Participants are given 2 minutes to complete as many items as possible.
9. *Letter-Number Sequencing (WAIS-III^UK^)* is conducted by testers reading increasingly long lists of letters and numbers, with participants asked to recall the list immediately afterwards by stating the numbers in numerical order and then the letters in alphabetical order.
10. *NART and WTAR* are often used to estimate ‘prior cognitive ability level’ since they tap word recognition and pronunciation, a cognitive ability very robust against age- and trauma- related cognitive decline. Each requires the pronunciation of 50 irregular words.
11. *Verbal Fluency* tests executive function. Participants are asked to list as many words as they beginning with C, and then F, and then L, with 1 minute for each letter. Here we used a total score across letters.
12. *Reaction Time* tests speed of processing. Here we use both simple and 4-choice mean reaction time scores. In the simple task which had 20 trials, participants had to press a 0 key as quickly as possible when presented with a 0 on screen. In the 4-choice task, which had 40 trials, participants are presented with a 1, 2, 3 or 4 on screen, and have to press the corresponding button (labelled 1, 2, 3 and 4) as quickly as possible.
13. *Inspection Time* tests efficiency of visual discrimination. It is a forced-choice, two-alternative psychophysical task using the method of constant stimuli. In each of the 150 trials, participants are presented with two parallel vertical lines of very different lengths and, without time pressure, are asked to select which of the lines is longer. Stimulus durations range from 6 ms to 200 ms and stimuli were backward masked immediately after presentation.
