# Supplementary material for: Brain White Matter Tract Integrity and Cognitive Abilities in Community-Dwelling Older People: The Lothian Birth Cohort, 1936
Source: Neuropsychology. 2013 Aug 12;27(5):595–607. doi: 10.1037/a0033354 (PMC3780714; doi:10.1037/a0033354)
Supplement: Supplementary file 2 [file NEU-NEU2-Booth20121387-R-S2.doc]

**Supplementary Material B**

**Table B1**: Factor loading matrix for exploratory factor analysis of the 18 cognitive tests.

|  | **Knowledge** | **Verbal Declarative Memory** | **Processing Speed** | **Non-Verbal Reasoning** |
| --- | --- | --- | --- | --- |
| *Eigenvalue* | 6.19 | 1.95 | 1.26 | 1.23 |
|  |  |  |  |  |
| Wechsler Test of Adult Reading | **0.94** | 0.03 | 0.01 | 0.03 |
| National Adult Reading Tests | **0.89** | 0.08 | 0.03 | 0.01 |
| Verbal Fluency Total Score | **0.31** | 0.00 | 0.29 | 0.07 |
| Logical Memory Delayed Recall | 0.02 | **0.92** | 0.02 | 0.03 |
| Logical Memory Immediate Recall | 0.05 | **0.91** | 0.02 | 0.00 |
| Verbal Paired Associates 2^nd^ Recall | 0.11 | **0.42** | 0.08 | 0.11 |
| Verbal Paired Associates 1^st^ Recall | 0.14 | **0.34** | 0.01 | 0.12 |
| Choice Reaction Time | 0.04 | -0.05 | **-0.80** | 0.01 |
| Digit Symbol | 0.17 | 0.06 | **0.58** | 0.16 |
| Simple Reaction Time | 0.02 | -0.02 | **-0.52** | 0.05 |
| Symbol Search | 0.12 | -0.01 | **0.50** | 0.26 |
| Inspection Time Total Score | 0.01 | 0.01 | **0.40** | 0.17 |
| Spatial Span Backwards | -0.11 | 0.03 | 0.09 | **0.60** |
| Block Design | 0.16 | -0.02 | 0.06 | **0.60** |
| Spatial Span Forwards | -0.12 | 0.03 | 0.09 | **0.55** |
| Matrix Reasoning | 0.16 | 0.11 | -0.02 | **0.53** |
| Digit Span Backwards | 0.19 | 0.10 | 0.04 | **0.39** |
| Letter-Number Sequencing | 0.18 | 0.13 | 0.21 | **0.35** |
|  |  |  |  |  |

Note: All values >0.30 are displayed in bold as this was used as the cut-off for salience of factor loadings
